# A One-Year longitudinal study on Surrender to God assessed during addiction treatment

**DOI:** 10.1016/j.abrep.2026.100693

**Published:** 2026-03-26

**Authors:** Henk-Jan Seesink, Cis Vrijmoeth, Brian D. Ostafin, Hanneke Schaap-Jonker, Reinout W. Wiers

**Affiliations:** a De Hoop, the Netherlands; b Addiction Development and Psychopathology (ADAPT)-lab, Department of Psychology, University of Amsterdam, the Netherlands; c Centre for Research and Innovation in Christian Mental Health Care, Eleos/De Hoop, the Netherlands; d Experimental Psychopathology and Clinical Psychology, Department of Psychology, University of Groningen, the Netherlands; e Faculty of Social Sciences and Humanities, School of Religion and Theology, Vrije Universiteit Amsterdam, the Netherlands

**Keywords:** Religion, Substance use disorder, Implicit association test, Surrender, Meaning in life

## Abstract

This study tested whether Surrender to God (StG), assessed explicitly (self-report) and implicitly (implicit association task), was a predictor of recovery characterized by reduced risk of relapse, reduced Substance Use Disorder (SUD) symptoms, and increased meaning in life. Longitudinal data (*N* = 177) were collected during treatment at a Christian inpatient SUD treatment facility, with follow-ups at one, six, and twelve months after treatment discharge. We used generalized estimating equations (GEE) analyses with the binary outcome relapse to estimate predictive relationships of explicit and implicit StG after treatment. Additionally, linear mixed model analyses (LMM) were conducted concerning SUD symptoms and meaning in life, with either implicit or explicit StG scores as predictors. The analyses were controlled for baseline scores of SUD symptoms and meaning in life including Bonferroni-Holm correction. Implicit StG predicted decreased odds of relapse (Odds ratio = 0.371, *p* = 0.001) over the course of one year but not at the one-month follow-up assessment. The reversed pattern was found for explicit StG, which predicted lower chances of relapse up to one month after treatment (Odds ratio = 0.962, *p* = 0.011) but not at 6 or 12-months assessments. Neither implicit nor explicit StG predicted follow-up SUD symptoms or meaning in life. These results suggest that explicit StG may be useful in predicting short-term relapse, and implicit StG may be useful in predicting longer-term relapse. In the context of religion-based recovery, theory and clinical practice may benefit from including explicit and implicit measures concerning religion and spirituality, such as StG.

## Highlights

• Treatment outcomes are examined using both explicit and implicit measures.
• The study addresses patients experience of surrender to God (StG).
• StG predicted lower relapse risk, even after controlling for meaning in life.
• Abstinence was linked with explicit StG (short-term) and implicit StG (long-term)

## 1 INTRODUCTION

Patients with substance use disorders (SUD) are more likely to face a life of disrupted social relationships, mental health problems, joblessness, education and housing problems, crime, and suicidal behaviour (Armoon et al., 2021, Dekkers et al., 2020, Flensborg-Madsen et al., 2009, Kõlves et al., 2017, Martinelli et al., 2020). Given these stark costs of SUD, research is needed regarding the processes that contribute to recovery. In general, absence of use and remission of SUD symptoms are important indicators of recovery. However, more recent conceptualizations of recovery include growth processes such as values of integrity (e.g., honesty towards oneself, taking responsibility) and helping others, more meaningful coping concerning stressful life events, and learning to enjoy life (Zemore et al., 2023). This study examines religiosity as a predictor of recovery and follows the broader perspective of recovery (Martinelli et al., 2020, Witkiewitz et al., 2020) by including both meaning in life and SUD behaviour as outcome variables.

Regarding recovery predictors, past research has given increased attention to spirituality and religiousness. Spirituality can be understood as a distinctive universal human experience of the search for what is perceived to be sacred (Cook, 2004, Miller, 1998, Pargament et al., 2013), while religiousness is seen as the search for significance within the context of established institutions facilitating spirituality (Pargament et al., 2013). These concepts have been shown to be relevant for recovery in the experience of patients (Kaskutas et al., 2014), to be inversely related to the prevalence and severity of SUD (Bonelli and Koenig, 2013, Walton-Moss et al., 2013), and to be an effective ingredient in SUD treatment (Hai et al., 2019, Heinz et al., 2007, Robinson et al., 2007, Schoenthaler et al., 2015, Zemore, 2007). Comprehensive models have proposed that religious coping strategies could increase well-being (Davis et al., 2022) and mental health (Koenig et al., 2023), which may also be relevant in SUD by providing a buffer against more negative emotions, stress and craving (Stellern et al., 2023, Swan et al., 2020, Wemm et al., 2019).

One potentially relevant religious coping strategy in SUD recovery is surrender to God (StG) (Cole and Pargament, 2004, Dyslin, 2008, James, 1902, Reinert, 1997, Tiebout, 1953). StG is a specific religious coping strategy that involves relinquishing one's desires and actions to follow what one believes to be God's will (Clements & Ermakova, 2012). When considered as a form of religious commitment (Clements et al., 2015), StG can lead to delayed gratification mediated by a future time orientation (Carter et al., 2012). As episodic future thinking can decrease the relative value of SUD reinforcers (Bickel et al., 2023), in contrast to negative repetitive thinking (Devynck et al., 2019, Hamonniere et al., 2020), StG could reduce SUD behaviour by a process described as humble detachment (Knabb et al., 2018). It refers to letting go of preoccupation with SUD reinforcers (detachment) and prioritising a transcendent awareness of God's active, loving presence over other needs (humility), which leads to less negative, repetitive thinking (Knabb et al., 2018) and greater tolerance of uncertainty (Knabb et al., 2017). StG may reduce negative emotions and craving, because it reduces worrying by transferring the cognitive, behavioural, and emotional exertion that comes with SUD problems from the self to God. Previous research has associated StG with less substance use (Hedrick et al., 2023), craving (Seesink et al., 2025), anxiety, depression, increased meaning in life (Seesink et al., 2024, Seesink et al., 2025, Wong-McDonald and Gorsuch, 2000), and the experience of being actively supported by God (Seesink et al., 2024). However, despite these findings and its inclusion in spiritual recovery programs (Dyslin, 2008), to our knowledge, an overarching theory and longitudinal evidence that StG promotes recovery are lacking. Therefore, this study examined whether StG relates to better recovery outcomes after treatment.

In addition to an explicit self-report measure (Seesink et al., 2024), this study also assessed StG with an implicit or indirect measure (De Houwer, 2006), the implicit association task (Seesink et al., 2025). Given that implicit measures are proposed to rely relatively more on automatic processes (De Houwer et al., 2009), an implicit measure may be well suited to predicting substance behaviour that feels difficult to control (Ostafin et al., 2014, Wolff et al., 2015; for review, see Lindgren et al., 2017).

Based on the research above, our primary hypotheses were that implicit and explicit StG, as measured during treatment for SUD, would be related to (1) less chance of relapse, (2) lower levels of SUD symptoms and (3) greater meaning in life at follow-up (i.e., one month, six months, and twelve months after clinical treatment).

## 2 METHODS

### 2.1 Participants and procedure

**Fig. 1:**
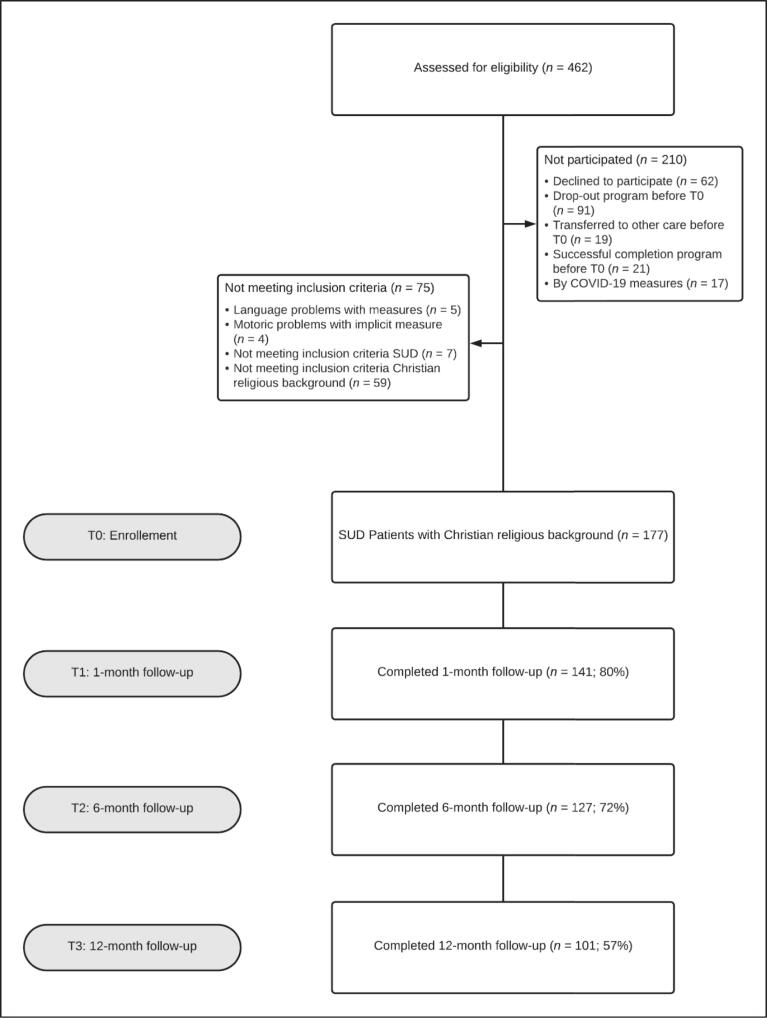
Flowchart of the longitudinal study. *Note*. T0 = Baseline; T1 = 1 month follow-up; T2 = 6 month follow-up; T3 = 12 month follow-up.

At the start of a 3-month inpatient program, 462 patients with SUD were approached to participate in this study at a government-accredited Christian addiction and mental health clinic in Dordrecht, the Netherlands (see Fig. 1). A multidisciplinary team of social workers, psychologists, psychiatrists, and doctors specializing in addiction medicine provided, in line with Dutch guidelines, disorder-specific treatment based on cognitive behavioural therapy for addiction (Schippers et al., 2014) and schema therapy for comorbid personality disorders (Young et al., 2003). If necessary, additional treatment with pharmacotherapy or trauma therapy was included. Patients were free to participate in optional, non-governmentally funded religious activities (e.g., prayer meetings) and to choose whether to connect their faith and spirituality to treatment goals. For further information on the treatment, see Supplemental Material A.

Prior to participating, patients were informed about the study and provided informed consent. The study was ethically approved by the ethics committee at the University of Amsterdam (registration ID: 2017-DP-7969) and is preregistered at https://www.aspredicted.org (#89523). The baseline assessment data was also used in a previous study (Seesink et al., 2025).

After the initial approach, 62 patients declined to participate, and 17 patients were unable to participate due to COVID-19. Others did not complete the study because of early treatment completion (*n* = 21), drop-out (*n* = 91), transfer to other care (*n* = 19) or no SUD as a primary DSM-5 diagnosis (*n* = 7). Moreover, five patients could not take the measures because of insufficient language fluency, and four participants had hand-motoric problems, which made it difficult to complete the implicit measure. As preregistered, non-Christian participants (n = 59) were excluded from the principal analyses but were not excluded from participation for reasons of preregistered exploratory analyses. Although many religions embrace StG (Cole & Pargament, 2004), the available sample was expected to include mainly Christians and, to a lesser extent, non-religious individuals, given the treatment context. Therefore, the results concerning StG were expected to be a valid predictor of treatment outcomes, mainly among Christian patients, as it is not likely to influence non-religious individuals. During treatment, at the beginning of the second month of clinical care, 177 Christian patients agreed to participate and started with the first assessment of this study (T0). Follow-up assessments were conducted by phone. One month after the treatment, 141 participants replied in the first follow-up (T1; 80%), followed by 127 replies after six months (T2; 72%) and 101 replies after twelve months (T3; 57%). All instruments described below were administered in the first assessment (T0). In the follow-up assessments (T1 to T3), the administration was limited to the outcome variables (relapse, SUD symptoms, and meaning in life).

Table 1 presents the background characteristics across all moments of measurement. At baseline, the primary SUD included alcohol (*n* = 86; 49%), cocaine (*n* = 45; 25%), cannabis (*n* = 19; 11%), opioids (*n* = 14; 8%), amphetamine (*n* = 11; 6%), and sedative, hypnotic or anxiolytic (*n* = 2; 1%) use disorder.

**Table 1:** Background characteristics of the patients at baseline, 1-, 6-, and 12-month follow-up.

	T0	T1	T2	T3
	(*N* = 177)	(*N* = 141)	(*N* = 127)	(*N* = 101)
Mean age	39.73	39.72	39.82	39.44
Gender				
Male	135 (76%)	105 (75%)	98 (77%)	76 (75%)
Female	42 (24%)	36 (25%)	29 (23%)	25 (25%)
Comorbid smoking	76 (43%)	60 (43%)	55 (43%)	41 (41%)
Comorbid SUD (no tobacco)	92 (52%)	69 (49%)	60 (47%)	52 (52%)
Comorbid DSM-5 disorder	138 (78%)	111 (79%)	101 (80%)	72 (71%)

*Note*. T0 = Baseline; T1 = 1 month follow-up; T2 = 6 month follow-up; T3 = 12 month follow-up.

### 2.2 Measures

#### 2.2.1 Explicit StG

Self-reported StG was measured only at baseline with the Dutch Surrender to God Scale (Seesink et al., 2024), a translation of the Surrender Scale (Wong-McDonald & Gorsuch, 2000). Participants answered twelve statements (score range: 12–60) on a five-point Likert scale, ranging from 1 (strongly disagree) to 5 (strongly agree). An example statement is ‘*I will select God's solution to a problem even if it requires self-sacrifice from me*’. Cronbach’s alpha was 0.97.

#### 2.2.2 Implicit StG

**Fig. 2:**
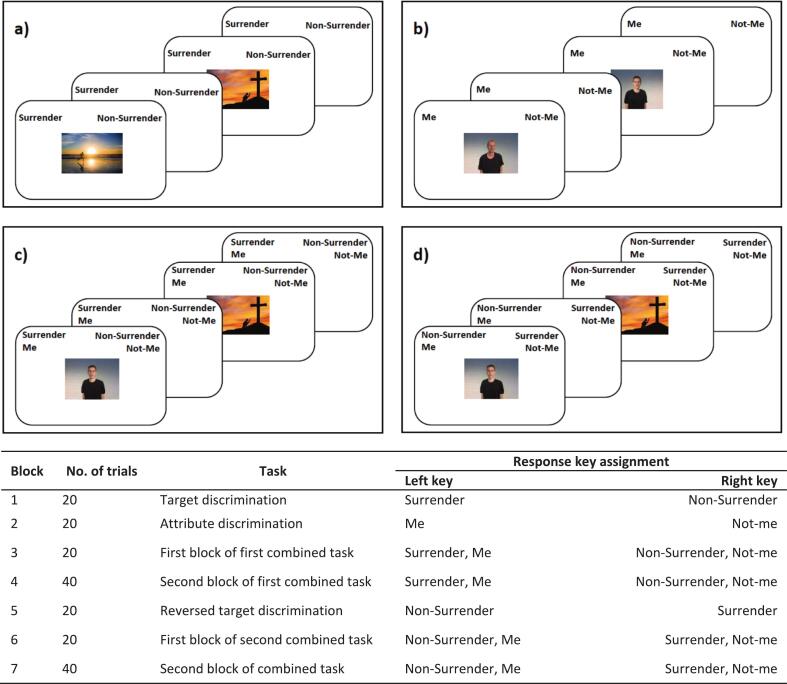
Task sequence and stimuli of the surrender-IAT to measure the implicit identity concept of surrender to God. *Note*. An overview of the four different blocks of the surrender-IAT. a) Categorizing pictures of surrender (e.g., a man kneeling before the cross during sunset) and non-surrender (e.g., a man jogging during sunset), block 1 & 5; b) Categorizing pictures of the participant (made in the lab) and not-me (non-participants controlled by gender and ethnic background), block 2; c) Congruent mapping of the combined task, block 3 & 4; d) Incongruent mapping of the combined task, block 6 & 7.

The Implicit Association Test (IAT) for surrender (Seesink et al., 2025) was administered at baseline to assess relatively spontaneous or automatic processes related to StG. The methods are shown in Fig. 2 and described in detail in Supplemental Material B. Following the standard test procedure, participants had to sort picture combinations of the constructs *Surrender to God* vs. *Non-surrender to God* and *Me* vs. *Not me* (Seesink et al., 2025), which resulted in the IAT-D score. The IAT-D scores could range between −2 and 2. In line with Perugini and colleagues (2010), the same sequence of blocks was maintained for all participants, starting with the compatible block (*Surrender to God* and *Me*) first. The measure was scored such that larger values indicate more StG. The split-half reliability was modest (*r* = 0.58).

#### 2.2.3 Relapse

Following the protocol ‘Deutsche Gesellschaft für Suchtforschung, Standard-4′ of the German Addiction Society (DGSS-4; cf. Eberl et al., 2013, Wiers et al., 2011), the preferred outcome was related to (1) no relapse at all or (2) a single lapse shorter than three days, ended by the patient without further negative consequences. Relapse (or return to use) was categorized as relapse or death, 'no information', or refusal to participate at follow-up (which was done by phone).

#### 2.2.4 SUD symptoms

SUD symptom severity levels were assessed with the Leeds Dependence Questionnaire at baseline and follow-up assessments. The 10-item self-report questionnaire (e.g., *Do you find it difficult to cope with life without drink or drugs?*) was used to monitor treatment outcome and assess substance dependencies (Raistrick et al., 1994), with a rating scale ranging from 0 (Never) to 3 (Nearly always). Higher sum scores determined a higher level of dependence (score range: 0–30). Across the assessments, Cronbach alphas were ≥ 0.89.

#### 2.2.5 Meaning in life

Meaning in life covers the experience that one’s life makes sense, matters, and is driven by valued goals, which was measured at baseline and all follow-ups by the total score of the Multidimensional Existential Meaning Scale (George & Park, 2017). The measure is a 15-item self-report questionnaire (score range: 15–105) with three subscales: comprehension (e.g., *My life makes sense*), purpose (e.g., *I have aims in my life that are worth striving for*), and mattering (e.g., *I am certain that my life is of importance*). All responses are rated on a 7-point scale, ranging from 1 (very strongly disagree) to 7 (very strongly agree). Cronbach alphas were ≥ 0.92.

### 2.3 Data analysis

In order to examine the predictive effects of baseline explicit and implicit StG on relapse over time, we performed two generalized estimating equations (GEE) analyses. For the effects on SUD symptoms and meaning in life over time a total of four linear mixed models (LMM) were conducted.^1^ For, relapse – which is a binary outcome measure – a GEE analysis was chosen as a logistic mixed model analyses may provide an overestimation of the effect estimate (Twisk et al., 2017) and because previous research suggested this analysis for addiction-related behaviour (e.g., Homish et al., 2010, Lee et al., 2007). The baseline scores of SUD symptoms and meaning in life were included as covariates because they were expected to relate to recovery. For instance, meaning in life may predict recovery because of the meaning or importance of addiction-related reinforcers (such as craving or associated rewards) becomes less central or dominant (Ostafin & Näther, 2025), explaining the evidence of an inverse relation between meaning in life and SUD (Krentzman et al., 2017, Roos et al., 2015).

All analyses were performed with SPSS version 25. For both GEE analyses, an exchangeable correlation structure was chosen. The models included relapse over time as an outcome measure and controlled for the baseline level of SUD symptoms and meaning in life. The final four LMM models included SUD symptoms or meaning in life as the outcome measure at each follow-up and baseline SUD symptoms and meaning in life as covariates to account for differences at the start of treatment.^2^ Correction for multiple testing (i.e., six tests) was done with the Bonferroni-Holm method (*p*-values ranged from 0.007 to 0.025). For all analyses, we first present the pre-registered overall effect (average over follow-up) and, when this was significant, post-hoc analyses on the effect at the separate follow-up assessments.

Prior to the main analyses, we performed a power analysis^3^ and checked for outliers and assumptions. Because of possible heteroscedasticity, the SUD symptoms were log transformed with the formula log_10_(x + 1). An error in the administration procedure resulted in eight missing values on the meaning in life measure (seven at baseline and one at 12-month follow-up). Therefore, before the primary analyses, we used a simple imputation procedure in which the averages of the scale scores replaced the missing values. Also, due to technical difficulties, seven participants could not provide scores for the implicit measure of StG and, therefore, could not be included in further analyses concerning implicit StG.

## 3 RESULTS

### 3.1 Descriptive statistics

**Fig. 3:**
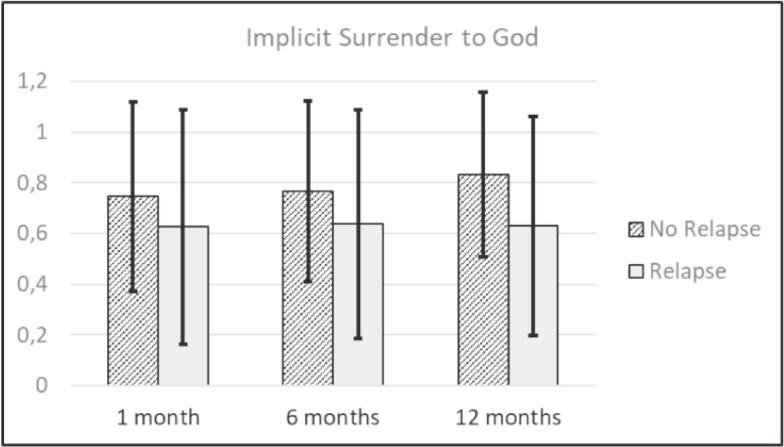
Average implicit Surrender to God scores related to relapse after treatment. *Note*. The bars above show the average baseline scores on implicit surrender to God sorted by participants with no relapse or relapse during the one-month, six-month, or 12-month follow-up. The bars represent standard deviations. A higher average score represents a stronger association of ‘surrender to God’ and ‘Me’.

**Fig. 4:**
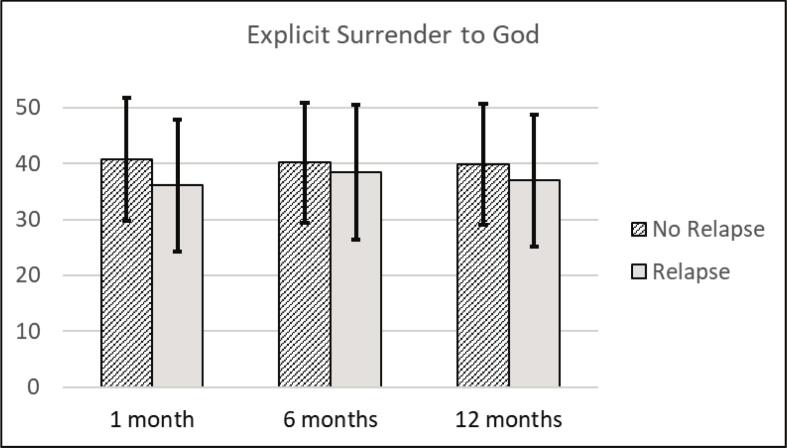
Average explicit Surrender to God scores related to relapse after treatment. *Note*. The bars above show the average baseline scores on explicit surrender to God sorted by participants with no relapse or relapse during the one-month, six-month, or 12-month follow-up. The bars represent standard deviations.

Table 2 shows the average baseline scores of explicit and implicit StG and the average scores of SUD symptoms and meaning in life at all time points. The correlation coefficients of baseline measures are found in Supplemental Material C. As preregistered, for the analysis of relapse, all participants we could not reach were still included in the analysis and characterized as relapsed. As a result, the percentages of participants without relapse shifted from 91% (only reached participants) to 72% (all participants) at one-month follow-up (no relapse: *n* = 128; relapse: *n* = 13; no information: *n* = 36), from 80% to 58% at six-month follow-up (no relapse: *n* = 102; relapse: *n* = 25; no information: *n* = 50) and from 70% to 40% at 12-month follow-up (no relapse: *n* = 71; relapse: *n* = 30; no information: *n* = 76). Fig. 3, Fig. 4 present the participants' average scores, sorted by relapse and no relapse, during different follow-up assessments for implicit StG and explicit StG, respectively.

**Table 2:** Descriptive statistics.

	*M*	*SD*	Min	Max	*N*
Explicit Surrender to God	39.20	11.38	12	60	177
Implicit Surrender to God	0.71	0.40	− 0.49	1.40	170
SUD symptoms					
Baseline	8.62	7.68	0	30	177
1 month	4.27	6.18	0	30	141
6 months	5.29	6.92	0	30	127
12 months	5.57	7.09	0	30	101
Meaning in life					
Baseline	69.53	17.67	30	101	177
1 month	75.77	18.29	19	105	141
6 months	76.13	18.01	25	105	127
12 months	77.65	17.30	24	105	101

*Note*. SUD = Substance Use Disorder.

### 3.2 Implicit and explicit StG and relapse

The results of the GEE analyses for the effect of implicit and explicit StG on relapse are shown in Table 3. As pre-registered, we were interested in the effects over time aggregated by the three follow-up measurements: one month, six months and twelve months. The baseline measure of implicit StG showed, with an average odds ratio of 0.371, to be a significant predictor of relapse. This result indicates that even after controlling for the baseline levels of SUD symptoms and meaning in life, the average odds of relapse over the course of one year was almost three times lower with one unit increase in implicit StG scores at baseline during clinical treatment.^4^ When looking at the different measurement moments, the effect was only significant at T2 and T3.^5^ The effect of explicit StG was only significant for relapse after one month but not overall (aggregating T1, T2 and T3).

**Table 3:** GEE analyses examining the effects of implicit and explicit Surrender to God on relapse over Time (N = 177).

	*B*	*SE B*	Odds ratio	*Wald* χ^2^	*p*	Confidence interval 95%^a^	
						Lower bound	Upper bound
**Implicit Surrender to God**							
*Overall effect (pre-registered)*							
T0 Meaning in Life	0.008	0.006	1.008	1.729	0.189	0.996	1.021
T0 SUD symptoms	0.020	0.015	1.020	1.731	0.188	0.990	1.050
T0 Implicit StG	−0.992	0.305	0.371	10.593	0.001^b^	0.204	0.674
*Effect at different follow-up (post-hoc)*							
T1	−0.784	0.455	0.456	2.975	0.085	0.187	1.113
T2	−0.893	0.421	0.410	4.503	0.034	0.180	0.934
T3	−1.506	0.442	0.222	11.629	0.001	0.093	0.527
**Explicit Surrender to God**							
*Overall effect (pre-registered)*							
T0 Meaning in Life	0.009	0.007	1.009	1.674	0.196	0.996	1.022
T0 SUD symptoms	0.020	0.015	1.020	1.792	0.181	0.991	1.051
T0 Explicit StG	−0.020	0.010	0.980	3.804	0.051	0.960	1.000
*Effect at different follow-up (post-hoc)*							
T1	−0.039	0.015	0.962	6.448	0.011	0.933	0.991
T2	−0.017	0.014	0.984	1.456	0.228	0.958	1.010
T3	−0.009	0.015	0.991	0.437	0.508	0.963	1.020

*Note*. A binary outcome relapse (= 1) and no relapse (= 0) is used. T0 = Baseline; T1 = 1 month follow-up; T2 = 6 month follow-up; T3 = 12 month follow-up; SUD = Substance use disorder; StG = Surrender to God;

a Wald confidence interval of 95% for Odds ratio;

b significant with Bonferroni-Holm correction.

### 3.3 Implicit and explicit StG and SUD symptoms

The results of the two LMM analyses examining the effect of implicit and explicit StG on the level of SUD symptoms are shown in Table 4. Neither implicit nor explicit StG significantly predicted the level of SUD symptoms. However, meaning in life was a predictor of lower SUD symptoms levels over the year in the analyses that also included baseline scores SUD symptoms and implicit StG as predictors.^6^

**Table 4:** LMM analyses examining the effects of implicit and explicit Surrender to God on SUD symptoms over time (N = 177).

	*B*	*SE B*	*t*	*p*	Confidence interval 95%	
					Lower bound	Upper bound
**Implicit Surrender to God**						
*Overall effect estimate (pre-registered)*						
T0 Meaning in life	−0.011	0.004	−2.898	0.004^a^	−0.019	−0.004
T0 SUD symptoms	0.020	0.009	1.810	0.072	−0.001	0.034
T0 Implicit StG	−0.147	0.194	−0.898	0.370	−0.400	0.211
**Explicit Surrender to God**						
*Overall effect estimate (pre-registered)*						
T0 Meaning in life	−0.009	0.004	−2.216	0.028	−0.016	−0.001
T0 SUD symptoms	0.016	0.009	1.980	0.049	0.001	0.034
T0 Explicit StG	−0.011	0.006	−1.771	0.079	−0.023	0.001

*Note*. The *B*, *SE* and confidence interval are presented with back-transformation using the formula: X=(10^(B)) −1. Models included a random intercept and random slope. T0 = Baseline; SUD = Substance use disorder; StG = Surrender to God;

a significant with Bonferroni-Holm correction.

### 3.4 Implicit and explicit StG and meaning in life

As presented in Table 5, the LMM analyses with meaning in life as the outcome showed that neither implicit nor explicit StG predicted meaning in life after treatment.

**Table 5:** LMM analyses examining the effects of implicit and explicit Surrender to God on meaning in life over time (N = 177).

	*B*	*SE B*	*t*	*p*	Confidence interval 95%	
					Lower bound	Upper bound
**Implicit Surrender to God**						
*Overall effect estimate (pre-registered)*						
T0 Meaning in life	0.612	0.057	10.718	<.001^a^	0.499	0.724
T0 SUD symptoms	0.083	0.126	0.507	0.507	−0.165	0.332
T0 Implicit StG	−0.761	2.595	−0.293	0.770	−5.887	4.367
**Explicit Surrender to God**						
*Overall effect estimate (pre-registered)*						
T0 Meaning in life	0.603	0.058	10.447	<.001^a^	0.489	0.717
T0 SUD symptoms	0.090	0.126	0.713	0.477	−0.159	0.339
T0 Explicit StG	−0.036	0.090	−0.403	0.687	−0.215	0.142

*Note*. Models included a random intercept. T0 = Baseline; SUD = Substance Use Disorder; StG = Surrender to God;

a significant with Bonferroni-Holm correction.

## 4 DISCUSSION

The study examined whether StG would predict recovery over the course of one year in Christian patients with SUD who received clinical treatment. The results showed mixed findings concerning a broader recovery perspective. On the one hand, we found that implicit StG predicted a lower probability of relapse, up to almost three times lower at one-year follow-up and even when controlling for baseline meaning in life and SUD symptoms. Explicit StG was also a significant predictor, but only at one-month follow-up. On the other hand, neither implicit nor explicit StG predicted fewer SUD symptoms or increased meaning in life. Further, analyses showed that baseline meaning in life was inversely related to follow-up SUD symptoms but not to relapse.

These results suggest that patients willing to StG may be less likely to relapse over the course of one year after treatment, even if they experience the same degree of SUD symptoms (e.g., dyscontrol) and meaning in life (e.g., purpose) as patients with less StG. Perhaps the inverse relationship between StG and relapse is explained by a shift in locus of control and future orientation, which increases resilience towards SUD behaviour even if the daily challenges of SUD recovery, such as SUD symptoms and the struggle for meaning in life, persist. A future time orientation could mediate between StG as religious commitment and delay gratification (Carter et al., 2012), which in turn reduces the excessive preference for substance reinforcers (Bickel et al., 2014). In addition, the locus of control on God in StG (Wong-McDonald & Gorsuch, 2004), shifting attention from mental fixations on one's needs to an orientation outside oneself regarding a benevolent God (Knabb et al., 2018), could increase tolerance of stress and uncertainty (Knabb et al., 2017). Both factors are known to contribute to SUD behaviour and treatment outcomes (Banducci et al., 2015, Garami et al., 2017, Vujanovic et al., 2022).

The elaborated intrusion theory of desire could explain resilience, as StG could inhibit the elaboration of intrusive thoughts, such as ‘I need alcohol’ (Kavanagh et al., 2005). Increased StG is associated with more religious activity (Seesink et al., 2024), leading to a context in which distracting religious external cues reduce the capacity to elaborate on intrusive thoughts. In addition, associated thoughts like ‘I need God to cope with addiction’ and anticipatory responses like the attribution of sensory experiences to God’s presence could stop further elaboration of the intrusive thought, ending the desire to drink alcohol. It may increase the chances of abstinence without affecting SUD symptoms and meaning in life. However, we remain cautious as the study did not measure intrusive thoughts.

Furthermore, the theory above suggests that affective and vivid images, such as the stimuli used in the implicit StG measure, can better predict craving during the elaboration process (Kavanagh et al., 2005). It could explain the results that implicit measures may be more beneficial in predicting SUD behaviour compared to explicit measures (Lindgren et al., 2017, Wolff et al., 2015). Remarkably, the effect of implicit StG was more pronounced at longer follow-up, while explicit StG only seemed to have an effect on the shorter term. This finding aligns with previous research on motives (Hofer and Chasiotis, 2022, McClelland et al., 1989). It suggests that while explicit measures may be relevant for immediate responses to structured situations (e.g., meeting friends who do not use substances), implicit measures also relate to spontaneous behaviour, such as unstructured situations (e.g., hearing bad news) that may trigger relapse (Huijding and De Jong, 2006, Roefs et al., 2011).

Despite StG’s potential to enhance resilience, no increase in meaning in life was found, suggesting that StG did not improve broader recovery beyond abstinence. Although religion is commonly cited as a source of meaning (King and Hicks, 2021, Mcmartin et al., 2020, Park, 2014, Spännäri and Laceulle, 2021, Zarzycka et al., 2020), and higher scores on StG have been associated with greater meaning in life (Seesink et al., 2024, Seesink et al., 2025, Wong-McDonald and Gorsuch, 2000), this finding suggests the need for further study of StG's relevance to other recovery domains.

### 4.1 Limitations

The study had some methodological limitations. For instance, although background characteristics remained similar during different assessments, and the response rate (57%) was higher than in earlier StG studies (Reinert, 1997; 43% in Study 2; 53% in Study 3), the twelve-month response rate was still relatively low, which may have affected the findings by non-response bias. Lower response rates may have impacted the relapse outcome because no contact was counted as relapse. Unfortunately, this method, which prevents missing data, was impossible for the follow-up data on SUD symptoms and meaning in life. The non-response may also have influenced SUD symptoms and meaning in life, as these outcomes were treated as missing values.

Based on the characteristics of the available sample, we limited our primary analyses to Christian patients. Therefore, the results are limited in generalizability to patients with other religious or life orientations. However, implicit StG remained a predictor of fewer relapse even when non-Christians were included in the analyses. The stimuli used in the surrender-IAT may also measure general aspects of surrender that could be relevant in non-Christian participants as well (e.g., humility). Nevertheless, the view that the spirituality of all adherents to all religions can be adequately evaluated by universal measures of StG could be misleading because views of StG and related values may vary between groups, suggesting the necessity of group-sensitive measures (Moberg, 2002).

Finally, as the study focused on StG during treatment, it cannot exclude the possibility that other factors at the start or after treatment (e.g., negative life events) may have influenced the results between StG and recovery outcomes. Multiple StG assessments during follow-up could improve understanding of changes in recovery outcomes.

## 5 Conclusion

In sum, the study provided evidence to support the hypothesis that StG may benefit addiction recovery, even outside the context of Twelve-Step treatment and even when controlling for meaning in life and SUD symptoms during treatment. Although StG was not a predictive factor for experiencing more meaning in life or (re)experiencing the physical and mental SUD symptoms, the results suggest a potential effect of StG on actual use after treatment (relapse). The prediction of relapse was more pronounced for implicit StG in the long term, whereas explicit StG seemed to have only a short-term effect. Future theories of addiction and clinical practice should address implicit and explicit measures of religion and spirituality.

## Primary Funding

The authors received no financial support for the research, authorship, and/or publication of this article.

## CRediT authorship contribution statement

**Henk-Jan Seesink:** Writing – review & editing, Writing – original draft, Methodology, Investigation, Formal analysis, Conceptualization. **Cis Vrijmoeth:** Writing – review & editing, Supervision, Formal analysis. **Brian D. Ostafin:** Writing – review & editing, Supervision, Methodology, Conceptualization. **Hanneke Schaap-Jonker:** Writing – review & editing, Supervision, Methodology, Conceptualization. **Reinout W. Wiers:** Writing – review & editing, Supervision, Software, Methodology, Conceptualization.

## Declaration of competing interest

Given their role as an Editorial Board member, Wiers R.W., had no involvement in the peer-review of this article and had no access to information regarding its peer-review. The other authors declare that they have no known competing financial interests or personal relationships that could have appeared to influence the work reported in this paper.

## Data Availability

The authors do not have permission to share data.

## Footnotes

1 These analyses control for correlated data due to repeated measures within the same individual: the LMM analyses by estimating variance between individuals (inclusion of random intercept and slope) and GEE analyses by directly modelling the correlation within individuals (i.e., choosing a correlation structure). For all four LMM analyses, we first checked whether to include a random slope next to a random intercept by comparing the −2log likelihood of a model with a random intercept and a model with a random intercept with an added random slope.

2 This concerns four LMM analyses. First, the dependent variable SUD symptoms at each follow-up with baseline scores for implicit StG, SUD symptoms, and meaning in life as independent variables. Second, the dependent variable is SUD symptoms at each follow-up, with baseline scores for explicit StG, SUD symptoms, and meaning in life as independent variables. Third, meaning in life at each follow-up as the dependent variable, with baseline scores of implicit StG, meaning in life, and SUD symptoms as independent variables. Fourth, meaning in life at each follow-up as the dependent variable, with baseline scores of explicit StG, meaning in life, and SUD symptoms as independent variables.

3 The required sample size of the LMM-analyses with G*Power (Faul et al., 2009) was based on a linear multiple regression, fixed model and R² deviation from zero (with three predictors; medium effect size of 0.15; alpha of 0.05; power of 0.80). This outcome was increased with 70%, as we expected an intraclass correlation of max. 0.70 because of repeated measurements within the same patient. For the GEE-analysis, the estimated relapse figures over time were conservatively set at 60% for participants who scored lower on StG and 40% for patients who scored higher on StG. Again with G*Power, we calculated the sample size for a logistic regression (two tailed, odds ratio of 0.446; Pr(Y=1|X=1)H0 = 0.5; alpha of 0.05; power of 0.80; R² other X of 0.20; normal distribution) and increased the outcome by 70%. The power analyses resulted in 131 and 133 participants respectively.

4 If the analyses were restricted to participants who were successfully contacted, the results would remain the same, except for the relapse outcome. Implicit StG presented a lower odds ratio for relapse, and explicit StG was significant over the course of one year (Supplemental Material D, Table 2). The pre-registered exploratory analyses with the sample including Christian, non-religious, and other religious backgrounds (*N* = 236) are presented in Supplemental Material F. Also, the pre-registered exploratory GEE analyses that included non-Christian patients revealed that implicit StG remained a predictor with a lower relapse odds ratio of 0.493 (see Supplemental Material F, Table 5).

5 Post-hoc, we analysed Pearson Correlations of the two StG measures and all relapse follow-up assessments to estimate a possible significant increase or decrease between the assessments. The strength between relapse T1 & T2 and relapse T2 & T3  were compared with a William’s test, resulting in a *z*-score and *p*-value. The StG measures showed no significant difference between relapse T1 and T2 and T2 and T3 (see Supplemental Material E).

6 For a comprehensive overview, we included the effect of meaning in life on the different measurement moments in the Supplemental Material (see Supplemental Material G).
