# Supplementary material for: A One-Year longitudinal study on Surrender to God assessed during addiction treatment
Source: Addict Behav Rep. 2026 Mar 26;23:100693. doi: 10.1016/j.abrep.2026.100693 (PMC13085016; doi:10.1016/j.abrep.2026.100693)
Supplement: Supplementary Data 1 [file mmc1.pdf]

Supplemental Material: A One-Year Longitudinal Study on Surrender to God Assessed During Addiction Treatment

Henk-Jan Seesink, Cis Vrijmoeth, Brian D. Ostafin, Hanneke Schaap-Jonker & Reinout W. Wiers

Content

Supplemental Material A: More information about the treatment program 1

Supplemental Material B: Description of the Surrender-Implicit Association Test (IAT)......................... 1

Supplemental Material C: Correlations Baseline measures 2

Supplemental Material D: Post-hoc analyses implicit and explicit StG and relapse outcome including only reached participants.. 3

Supplemental Material E: Post-hoc analyses of implicit and explicit StG correlations with all relapse follow-up assessments 3

Supplemental Material F: Pre-registered exploratory analysis with the sample including Christian, non-religious, and other religious backgrounds (*N* = 236) 4

Supplemental Material G: Pre-registered LMM analyses with post-hoc meaning in life effects at different time points... 7

Supplemental Material A: More information about the treatment program

The three-month Christian inpatient programme is open to people of all faiths. It is officially recognised by the government as an addiction care and psychiatric hospital and is financed by the basic health insurance coverage that every resident in the Netherlands has. The treatment does not follow a 12-step approach; instead, it uses cognitive-behavioural and schema therapy. Additionally, various aspects of the treatment provide patients with the option (no necessity) to address their spiritual and religious needs. As the institution develops treatment goals using a biopsychosocial and spiritual model of health, patients with SUD focus with their goals on four relationships: (1) with themselves, (2) with others, (3) with the environment, and (4) with God or another existential goal for people who do not believe in God.

Clinical observations in preparation for our research line showed that Surrender to God (StG) is an important topic in treatment. While practitioners do not include StG as a central or systematic focus of the treatment programme, they create space for it to emerge in practice through individualised goals and activities. Occasionally, StG becomes an explicit treatment goal (e.g., wanting to think more functionally, based on the realisation that God has other plans for the individual than drinking). More often, StG is implicitly addressed in relation to other goals (e.g., learning to forgive others because the patient feels God requires it). Patients can support these goals by voluntarily participating in elements such as prayer meetings, Biblical reflections at the start of the day, additional conversations with pastoral workers and spiritual carers, praise evenings, facilitating church visits, and organising conferences with speakers focused on themes relevant to SUD (e.g., forgiveness). These additional components are funded by donations. The practitioners involved—social workers, psychiatrists, nurses, doctors specialising in addiction medicine, and psychologists— do not follow a 12-step approach, but they can integrate aspects of patients' religion and spirituality into treatment.

Supplemental Material B: Description of the Surrender-Implicit Association Test (IAT)

The IAT (Greenwald et al., 1998; best practice: Greenwald et al., 2022) is a reaction-time task assumed to measure automatic (implicit) associations. The main idea: people respond faster when two concepts strongly associated in their mind share a response key, and more slowly when weakly associated concepts share a key. The IAT infers implicit associations from these speed differences. Figure 2 in the manuscript shows four categories. In our study, the target categories are Surrender (pictures associated with surrender to God) versus Non-surrender (pictures not associated with surrender but with matched valence and arousal). Attribute categories are Me (pictures of the participant) versus Not-me (pictures of an unknown person).

Participants sit at a computer and see one stimulus at a time. Each stimulus is a picture related to one of the categories. Participants categorize it as quickly and accurately as possible by pressing one of two response keys (e.g., left or right). Each response key is assigned to two categories at once. The task consists of several blocks, including practice blocks. In the practice blocks, participants learn to categorize the target concepts (Surrender vs. Non-surrender) and attribute concepts (Me vs. Not-me) separately.

In the critical combined blocks, target and attribute categories are systematically paired. In one block, called the congruent condition, Surrender pictures and Me pictures are assigned to the same response key, while Non-surrender pictures and Not-me pictures are assigned to the opposite key. In another block, the incongruent condition, these pairings are reversed: Surrender pictures and Not-me are paired on one key, while Non-surrender pictures and Me are paired on the other.

Reaction times and error rates are recorded for each trial. Implicit associations are inferred from differences in response speed between congruent and incongruent blocks. Faster responses are expected when Surrender and Me share a response key than when Surrender and Not-me do. This indicates a stronger implicit association between surrender to God and the self. A standardized IAT D-score is calculated for each participant (Greenwald et al., 2003). Higher positive values reflect a stronger implicit association between surrender to God and the individual.

Supplemental Material C: Correlations Baseline measures

Table 1

Pearson Correlations of all Baseline Measures (N = 177).

Measures	1	2	3	4
1. T0 Implicit StG	-			
2. T0 Explicit StG	.33***	-		
3. T0 Meaning in Life	.34***	.41***	-	
4. T0 SUD symptoms	-.08	-.09	-.25***	-

Note. T0 = Baseline; StG = Surrender to God; SUD = Substance Use Disorder. *$p \le .05$. **$p \le .01$. ***$p \le .001$ (two-tailed).

Supplemental Material D: Post-hoc analyses implicit and explicit StG and relapse outcome including only reached participants

Table 2

GEE analyses examining the effects of implicit and explicit Surrender to God on relapse over time with only reached participants

	B	*SE B*	Odds ratio	*Wald* χ^2	*p*	Confidence interval 95%[a]	
						Lower bound	Upper bound
**Implicit Surrender to God**							
Overall effect (pre-registered)							
T0 Meaning in Life	.008	.009	1.008	1.697	.347	.991	1.026
T0 SUD symptoms	.026	.020	1.026	1.697	.193	.987	1.066
T0 Implicit StG	-1.445	.434	.236	11.106	.001	.080	1.054
**Explicit Surrender to God**							
Overall effect (pre-registered)							
T0 Meaning in Life	.010	.009	1.010	1.258	.262	.992	1.028
T0 SUD symptoms	.023	.020	1.024	1.364	.243	.984	1.064
T0 Explicit StG	-.040	.014	.961	8.413	.004	.935	.987

Note. A binary outcome relapse (= 1) and no relapse (= 0) is used. T0 = Baseline; T1 = 1 month follow-up; T2 = 6 month follow-up; T3 = 12 month follow-up; SUD = Substance use disorder; StG = Surrender to God; [a]Wald confidence interval of 95% for Odds ratio.

Supplemental Material E: Post-hoc analyses of implicit and explicit StG correlations with all relapse follow-up assessments

Table 3

Pearson Correlations of StG and all relapse follow-up assessments (N = 177)

Measures	T1	T2	T3	Difference T1 & T2		Difference T2 & T3	
				z	*p*	*z*	*p*
T0 Implicit StG	-.132	-.159*	-.247***	.360	.360	1.047	.148
T0 Explicit StG	-184*	-.071	-.029	-1.534	.063	-.496	.310

Note. A binary outcome relapse (= 1) and no relapse (= 0) is used. T0 = Baseline; T1 = 1 month follow-up; T2 = 6 month follow-up; T3 = 12 month follow-up; StG = Surrender to God. *$p \leq .05$. **$p \leq .01$. ***$p \leq .001$ (two-tailed). A William's test is used to determine the difference between T1 & T2 and T2 & T3.

Supplemental Material F: Pre-registered exploratory analysis with the sample including Christian, non-religious, and other religious backgrounds (*N* = 236)

Table 4

Overall effect estimates of Generalized Estimating Equation analyses of explicit surrender to God with binary outcome relapse (= 1) and no relapse (= 0) over one month, six months, and twelve months with the sample including Christian, non-religious, and other religious backgrounds (N = 236)

	B	*SE B*	Odds ratio	*Wald* χ^2	*p*	Confidence interval 95%[a]	
						Lower bound	Upper bound
Overall effect estimate							
T0 Meaning in Life	.009	.006	1.009	2.670	.102	.998	1.020
T0 SUD symptoms	.018	.013	1.019	1.016	.156	.993	1.045
T0 Explicit surrender to God	-.007	.007	.993	7.199	.313	.978	1.007

Note. T0 = Baseline; T1 = 1 month follow-up; T2 = 6 month follow-up; T3 = 12 month follow-up; SUD = Substance use disorder; [a]Wald confidence interval of 95% for Odds ratio.

Table 5

Overall effect estimates of Generalized Estimating Equation analyses of implicit surrender to God with binary outcome relapse (= 1) and no relapse (= 0) over one month, six months, and twelve months with the sample including Christian, non-religious, and other religious backgrounds (N = 226)

	B	*SE B*	Odds ratio	*Wald* χ^2	*p*	Confidence interval 95%[a]	
						Lower bound	Upper bound
Overall effect estimate							
T0 Meaning in Life	.008	.005	1.008	2.225	.136	.998	1.018
T0 SUD symptoms	.018	.013	1.018	1.913	.167	.993	1.044
T0 Implicit surrender to God	-.708	.264	.493	7.199	.007	.294	.826

Note. T0 = Baseline; T1 = 1 month follow-up; T2 = 6 month follow-up; T3 = 12 month follow-up; SUD = Substance use disorder; [a]Wald confidence interval of 95% for Odds ratio. Due to technical difficulties, ten participants could not provide scores for the implicit measure of StG and, therefore, could not be included in the analyses.

Table 6

Overall effect estimates and effect estimates of different follow-up of the Linear Mixed Model analyses of explicit surrender to God with log-transformed SUD symptoms as dependent variable over one month, six months, and twelve months (N = 236)

	B	*SE B*	*t*	*p*	Confidence interval 95%	
					Lower bound	Upper bound
Overall effect estimate						
T0 Meaning in life	-.003	.001	-2.391	.018	-.007	-.001
T0 SUD symptoms	.009	.003	2.937	.004	.003	.016
T0 Explicit surrender to God	-.001	.002	-.766	.445	-.005	.002

Note. T0 = Baseline; T1 = 1 month follow-up; T2 = 6 month follow-up; T3 = 12 month follow-up; SUD = Substance use disorder. Different models were created: A naïve model (model 1), a naïve model with a random intercept (model 2), and a naïve model with a random intercept and a random slope (model 3). In the analyses Model 3 provided the best model fit.

Table 7

Overall effect estimates and effect estimates of different follow-up of the Linear Mixed Model analyses of implicit surrender to God with log-transformed SUD symptoms as dependent variable over one month, six months, and twelve months (N = 226)

	B	*SE B*	*t*	*p*	Confidence interval 95%	
					Lower bound	Upper bound
Overall effect estimate						
T0 Meaning in life	-.004	.001	-2.761	.006	-.006	-.001
T0 SUD symptoms	.009	.003	2.732	.007	.002	.015
T0 Implicit surrender to God	-.104	.060	-1.743	.083	-.222	.014

Note. T0 = Baseline; T1 = 1 month follow-up; T2 = 6 month follow-up; T3 = 12 month follow-up; SUD = Substance use disorder. Different models were created: A naïve model (model 1), a naïve model with a random intercept (model 2), and a naïve model with a random intercept and a random slope (model 3). In the analyses Model 3 provided the best model fit. Due to technical difficulties, ten participants could not provide scores for the implicit measure of StG and, therefore, could not be included in the analyses.

Table 8

Overall effect estimates and effect estimates of different follow-up of the Linear Mixed Model analyses of explicit surrender to God with meaning in life as dependent variable over one month, six months, and twelve months (N = 236)

	B	*SE B*	*t*	*p*	Confidence interval 95%	
					Lower bound	Upper bound
Overall effect estimate						
T0 Meaning in life	.650	.046	14.011	<.001	.558	.741
T0 SUD symptoms	.069	.107	.643	.521	-.142	.280
T0 explicit surrender to God	.017	061	.284	.777	-.04	.138

Note. T0 = Baseline; T1 = 1 month follow-up; T2 = 6 month follow-up; T3 = 12 month follow-up; SUD = Substance Use Disorder. Different models were created: A naïve model (model 1), a naïve model with a random intercept (model 2), and a naïve model with a random intercept and a random slope (model 3). In the analyses Model 3 provided the best model fit.

Table 9

Overall effect estimates and effect estimates of different follow-up of the Linear Mixed Model analyses of implicit surrender to God with meaning in life as dependent variable over one month, six months, and twelve months (N = 226)

	B	*SE B*	*t*	*p*	Confidence interval 95%	
					Lower bound	Upper bound
Overall effect estimate						
T0 Meaning in life	.667	.044	15.203	<.001	.580	.753
T0 SUD symptoms	.066	.107	.619	.536	-.144	.276
T0 Implicit surrender to God	-.268	2.000	-.134	.894	-4.210	3.675

Note. T0 = Baseline; T1 = 1 month follow-up; T2 = 6 month follow-up; T3 = 12 month follow-up; SUD = Substance Use Disorder. Different models were created: A naïve model (model 1), a naïve model with a random intercept (model 2), and a naïve model with a random intercept and a random slope (model 3). In the analyses Model 3 provided the best model fit. Due to technical difficulties, ten participants could not provide scores for the implicit measure of StG and, therefore, could not be included in the analyses.

Supplemental Material G: Pre-registered LMM analyses with post-hoc meaning in life effects at different time points

Table 10

LMM analyses examining the effects of implicit and explicit Surrender to God on SUD symptoms over time with meaning in life effects at different follow-up (N = 177)

	B	*SE B*	*t*	*p*	Confidence interval 95%	
					Lower bound	Upper bound
**Meaning in life and implicit StG**						
Overall effect estimate (pre-registered)						
T0 Meaning in life	-.011	.004	-2.898	.004[a]	-.019	-.004
T0 SUD symptoms	.020	.009	1.810	.072	-.001	.034
T0 Implicit StG	-.147	.194	-.898	.370	-.400	.211
Effect at different follow-up (post-hoc)						
T1	-.013	.005	-2.750	.006	-.023	-.004
T2	-.012	.005	-2.337	.020	-.021	-.002
T3	-.003	.005	-.490	.625	-.013	.008
**Meaning in life and explicit StG**						
Overall effect estimate (pre-registered)						
T0 Meaning in life	-.009	.004	-2.216	.028[b]	-.016	-.001
T0 SUD symptoms	.016	.009	1.980	.049[b]	.001	.034
T0 Explicit StG	-.011	.006	-1.771	.079	-.023	.001
Effect at different follow-up (post-hoc)						
T1	-.012	.005	-.997	.014	-.021	-.002
T2	-.007	.005	-.971	.126	-.017	.002
T3	-.008	.005	-.300	.877	-.011	.010

Note. The *B*, *SE* and confidence interval are presented with back-transformation using the formula: X=(10^(B)) -1. Models included a random intercept and random slobe. As only baseline meaning in life is significant, only meaning in life effects at different follow-up moments are presented and not of surrender to God. T0 = Baseline; T1 = 1 month follow-up; T2 = 6 month follow-up; T3 = 12 month follow-up; SUD = Substance use disorder; StG = Surrender to God; [a]significant with Bonferroni-Holm correction; [b] not significant with Bonferroni-Holm correction.

Table 11

LMM analyses examining the effects of implicit and explicit Surrender to God on meaning in life over time with meaning in life effects at different follow-up (N = 177)

	B	*SE B*	*t*	*p*	Confidence interval 95%	
					Lower bound	Upper bound
**Meaning in life and implicit StG**						
Overall effect estimate (pre-registered)						
T0 Meaning in life	.612	.057	10.718	<.001[a]	.499	.724
T0 SUD symptoms	.083	.126	.507	.507	-.165	.332
T0 Implicit StG	-.761	2.595	-.293	.770	-5.887	4.367
Effect at different follow-up (post-hoc)						
T1	.670	.069	9.739	<.001	.534	.805
T2	.609	.071	8.522	<.001	.468	.749
T3	.533	.078	6.847	<.001	.380	.686
**Meaning in life and explicit StG**						
Overall effect estimate (pre-registered)						
T0 Meaning in life	.603	.058	10.447	<.001[a]	.489	.717
T0 SUD symptoms	.090	.126	.713	.477	-.159	.339
T0 Explicit StG	-.036	.090	-.403	.687	-.215	.142
Effect at different follow-up (post-hoc)						
T1	.660	.069	9.596	<.001	.529	.796
T2	.603	.070	8.556	<.001	.464	.742
T3	.513	.078	6.579	<.001	.360	.666

Note. Models included a random intercept. T0 = Baseline; T1 = 1 month follow-up; T2 = 6 month follow-up; T3 = 12 month follow-up; SUD = Substance Use Disorder; StG = Surrender to God; [a]significant with Bonferroni-Holm correction.

References

Greenwald, A. G., Brendl, M., Cai, H., Cvencek, D., Dovidio, J. F., Friese, M., Hahn, A., Hehman, E., Hofmann, W., Hughes, S., Hussey, I., Jordan, C., Kirby, T. A., Lai, C. K., Lang, J. W. B., Lindgren, K. P., Maison, D., Ostafin, B. D., Rae, J. R., … Wiers, R. W. (2022). Best research practices for using the Implicit Association Test. *Behavior Research Methods, 54*, 1161–1180. https://doi.org/10.3758/s13428-021-01624-3/Published

Greenwald, A. G., Mcghee, D. E., & Schwartz, J. L. K. (1998). Measuring Individual Differences in Implicit Cognition: The Implicit Association Test. J*ournal of Personality and Soclal Psychology, 74*(6), 1464–1480.

Greenwald, A. G., Nosek, B. A., & Banaji, M. R. (2003). "Understanding and using the Implicit Association Test: I. An improved scoring algorithm": Correction to Greenwald et al. (2003). *Journal of Personality and Social Psychology, 85*(3), 481–481. https://doi.org/10.1037/h0087889
